# Characterizing Air Temperature Changes in the Tarim Basin over 1960–2012

**DOI:** 10.1371/journal.pone.0112231

**Published:** 2014-11-06

**Authors:** Dongmei Peng, Xiujun Wang, Chenyi Zhao, Xingren Wu, Fengqing Jiang, Pengxiang Chen

**Affiliations:** 1 State Key Laboratory of Desert and Oasis Ecology, Xinjiang Institute of Ecology and Geography, Chinese Academy of Sciences, Urumqi, Xinjiang, China; 2 Graduate University of Chinese Academy of Sciences, Beijing, China; 3 Earth System Science Interdisciplinary Center, University of Maryland, College Park, Maryland, United States of America; 4 IM System Group and Environmental Modeling Center, National Centers for Environmental Prediction, National Oceanic and Atmospheric Administration, College Park, Maryland, United States of America; 5 Xinjiang Meteorological Observatory, Urumqi, Xinjiang, China; 6 Information Centre of Xinjiang Xingnong-Net, Urumqi, China; University of Vigo, Spain

## Abstract

There has been evidence of warming rate varying largely over space and between seasons. However, little has been done to evaluate the spatial and temporal variability of air temperature in the Tarim Basin, northwest China. In this study, we collected daily air temperature from 19 meteorological stations for the period of 1960–2012, and analyzed annual mean temperature (AMT), the annual minimum (T_min_) and maximum temperature (T_max_), and mean temperatures of all twelve months and four seasons and their anomalies. Trend analyses, standard deviation of the detrended anomaly (SDDA) and correlations were carried out to characterize the spatial and temporal variability of various mean air temperatures. Our data showed that increasing trend was much greater in the T_min_ (0.55°C/10a) than in the AMT (0.25°C/10a) and T_max_ (0.12°C/10a), and the fluctuation followed the same order. There were large spatial variations in the increasing trends of both AMT (from −0.09 to 0.43 °C/10a) and T_min_ (from 0.15 to 1.12°C/10a). Correlation analyses indicated that AMT had a significantly linear relationship with T_min_ and the mean temperatures of four seasons. There were also pronounced changes in the monthly air temperature from November to March at decadal time scale. The seasonality (i.e., summer and winter difference) of air temperature was stronger during the period of 1960–1979 than over the recent three decades. Our preliminary analyses indicated that local environmental conditions (such as elevation) might be partly responsible for the spatial variability, and large scale climate phenomena might have influences on the temporal variability of air temperature in the Tarim Basin. In particular, there was a significant correlation between index of El Niño-Southern Oscillation (ENSO) and air temperature of May (P = 0.004), and between the index of Pacific Decadal Oscillation (PDO) and air temperature of July (P = 0.026) over the interannual to decadal time scales.

## Introduction

There have been numerous studies of temporal variations of air temperature at various spatial scales [1]–[5], which all have pointed to the fact that there has been a general warming trend in the global mean air temperature. Earlier studies indicated that the magnitude of warming in the Northern Hemisphere (0.30°C/10a) was more than double of the one (0.13°C/10a) in the Southern Hemisphere during 1977–2001 [6], [7].

There has been evidence of difference in warming trend over space and time in China. For example, the warming rate of air temperature was 0.25°C/10a for the period of 1951–2004 in China [8], but 0.35°C/10a in northwest China during the period of 1961–2006 [9], which were much higher than the global average. Over the past 50 years, an increase of air temperature with a linear tendency of 0.28°C/10a was observed in Xinjiang, which was lower than that for the northwest China [10]. A couple of studies showed that the warming in the Tarim Basin was only from 0.19 to 0.22°C/10a [11], [12]. These findings indicated that climate change in Xinjiang might have its own spatial and temporal characteristics due to its large extent and complex terrain.

Apart from the increasing trend in annual mean temperature (AMT), there has been evidence of differences in warming rate between seasons at various regional scales. An earlier study based on analyses of 726 stations' data (1951–2004) in China showed an increasing trend of 0.39°C/10a in winter, 0.28°C/10a in spring, 0.20°C/10a in autumn and only 0.15°C/10a in summer [8]. Some studies indicated that the increasing rate of individual season's temperature was even greater in northwest China, in particular in winter. For example, Wang et al. [13] reported a rate of 0.56°C/10a in winter, 0.35°C/10a in autumn, 0.26°C/10a in spring, and 0.22°C/10a in summer for the period of 1960–2005. A recent study by Li et al. [14] showed an increasing trend of 0.49, 0.36, 0.27 and 0.23°C/10a for winter, autumn, spring and summer, respectively, for the period of 1960–2010. These results imply that there are significant changes in the seasonality of air temperature in the vast arid/semi-arid regions. However, there were limited analyses for the Tarim Basin, which were based on data of 1958–2004 from six meteorological stations in the western section of the basin [15], showing an increasing trend of 0.4 and 0.3°C/10a in winter and autumn, respectively.

Large scale climate phenomena often have various impacts on local climate. For example, El Niño-Southern Oscillation (ENSO) has widespread influence on the climate of north America and east Asia. An early study indicated that ENSO had significant effects on precipitation and temperature in the basins in Xinjiang [16]. However, other studies suggested that ENSO had no significant effect on the annual temperature in the Tarim basin [15], [17]. On the other hand, there was evidence of interannual to decadal variations in air temperature in the Tarim Basin [17]–[19]. However, little is done to evaluate the temporal variability of air temperature change and the underlying mechanisms.

While there have been some studies of the climate changes in the Tarim Basin [12], [15], [20], there is a lack of consistence in findings, primarily due to the difference in the studying period (thus difference in dataset). In addition, detailed analyses of the spatial and temporal variations in air temperature have been lacking for the Tarim Basin, which hampers our understanding of the climate change at local to regional scales. To address this issue, we carry out a detailed study, using the latest dataset covering the period of 1958–2012. Our approaches include anomalies, trend analyses, and fluctuation and correlation analyses. The objective of this study is to investigate the spatial and temporal variability in air temperature at seasonal to decadal time scales, and to explore the possible mechanisms responsible for these changes.

## Materials and Methods

### Description of study area

**Figure 1:**
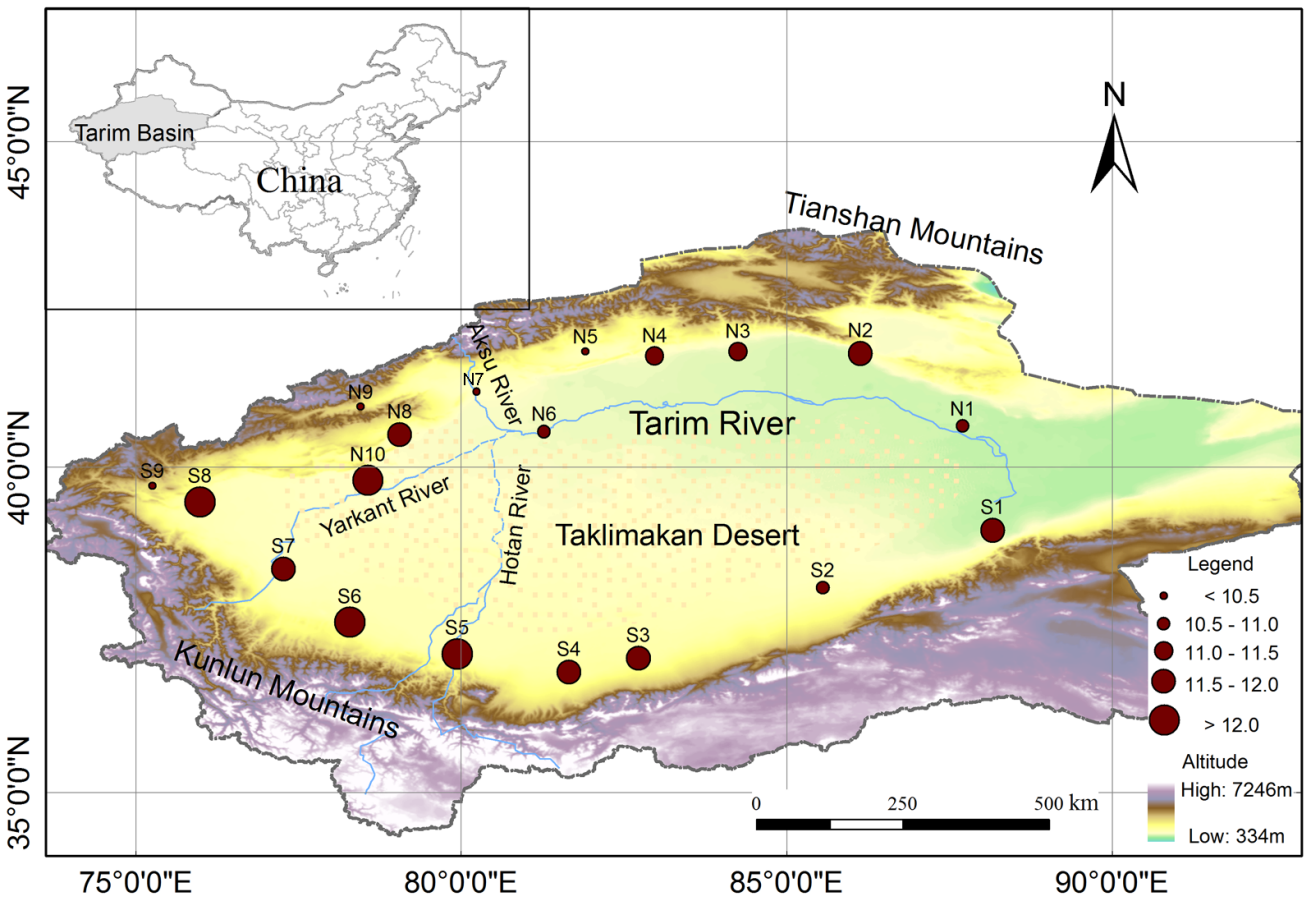
Topographic map with the locations of the meteorological stations in Tarim Basin and climatology AMT for the period of 1960–2012.

The Tarim Basin is located in the southern part of the Xinjiang Autonomous Region, Northwestern China, which is surrounded by high mountains (i.e., Tianshan Mountain and Kunlun Mountain) (Figure 1). The main river system, the Tarim River, consists of the Yarkant River, Aksu River, Hotan River, and Kaidu River. Rivers are primarily fed by snow and glacier melting waters from the mountains. The Tarim Basin (with a total area of 1.02× 10^6^ km^2^) is divided into the mountains (47%, with elevation of 4000–6000 m), plains (22%, with elevation of 800–1400 m), and deserts (31%, with elevation of 1200–1500 m in the western and southern parts, and 800–1000 m in the eastern and northern parts). The climate is typical continental, with an average annual temperature of 10.6–11.5°C and precipitation of 17.4–42.0 mm [21].

### Data sources and analyses

#### Data sources

We obtained daily mean air temperature data of 19 meteorological stations for the period of 1960–2012 from the National Climatic Center, China Meteorological Administration (http://cmdp.ncc.cma.gov.cn/cn/index.htm). These stations have the most completed dataset with acceptable quality control [22]. Figure 1 shows the spatial distribution of these stations and AMT over the period of 1960–2012. As shown in Figure 1, AMT varies from 6.57°C to 12.75°C, with higher values in the southwest stations than in the northeast stations. However, the lowest AMT was found at the stations with higher elevation in the northwest section.

We obtained three common indices of large-scale climate variability, i.e., the Southern Oscillation Index (SOI), the Pacific Decadal Oscillation (PDO), and the Indian Ocean Dipole Mode Index (DMI). The SOI data were obtained from the Climate Prediction Center (CPC) of the National Weather Service, U.S. (http://www.cpc.ncep.noaa.gov/), PDO index from N. Mantua (http://jisao.washington.edu/pdo/), and DMI from the web site (http://www.jamstec.go.jp/frcgc/research/d1/iod/).

#### Data analyses

We used daily mean temperature to derive the minimum (T_min_) and maximum (T_max_) temperature, and average temperatures for all twelve months, spring (March-May), summer (June-August), autumn (September-November) and winter (December-February) for each year. Our analyses included anomalies, trend analysis, fluctuation analysis, and correlation. All calculations were performed using the SPSS 19.

Temperature anomaly (TA) was determined by removing the mean value (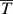) that was calculated for the period of 1960–2012:
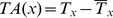
(1)


where *x* represented any temperature index (e.g., T_min_ and T_max_).

Trend analysis was carried out by linear regression that has been a common method to determine the long-term changing trend of air temperature [23]: 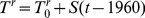
(2)


where T^r^ and 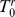 were the predicted temperature for year *t* and 1960, respectively. The slope *S* represented the trend of temperature increase, and the strength and significance of the linear increase were indicated by the regression coefficient (*R*) and P value. In general, when the *P* value was smaller than 0.05, the trend was significant.

Detrended fluctuation analysis (DFA) has proven useful in revealing the extent of long range correlations in time series [24], [25]. We carried out a similar analysis but with a single time window to determine the extent of fluctuation in various temperature indices, which was the standard deviation of the detrended time series of anomaly (SDDA) and calculated as:
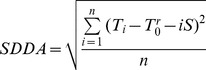
(3)


where *T_i_* was the *i*th temperature, and *n* the number of data points. In general, the smaller the SDDA value, the smaller the fluctuation is.

Correlation analyses were carried out to examine the relationships between AMT and other temperatures and the potential impacts of climate phenomena over the period of 1961–2013. We used annual means of PDO and DMI indices and the mean value of SOI over winter. We evaluated the relationship between various temperature anomalies with ENSO, DMI and the PDO indices. We also conducted linear and nonlinear regression analyses to examine the relationship between the change rates of AMT or T_min_ and elevation, latitude and longitude. In addition, multi-regressions were carried out to evaluate the influences of local factors and climate phenomena.

Correlation coefficient (*R*) was used to indicate the strength and direction of the relationship between the observation (x) and prediction (y), which was defined as the covariance of the variables divided by the product of their standard deviations:
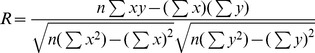
(4)


where *n* was the number of pairs of data. The value of R ranged from −1 to +1. The + and – signs indicated positive and negative linear correlations, respectively. The same as the regression analysis, the correlation coefficient *R* was assessed by the *P* value. When *P* was smaller than 0.05, the two variables were considered to have a significant correlation.

## Results

### Temperature change trend and fluctuation

**Figure 2:**
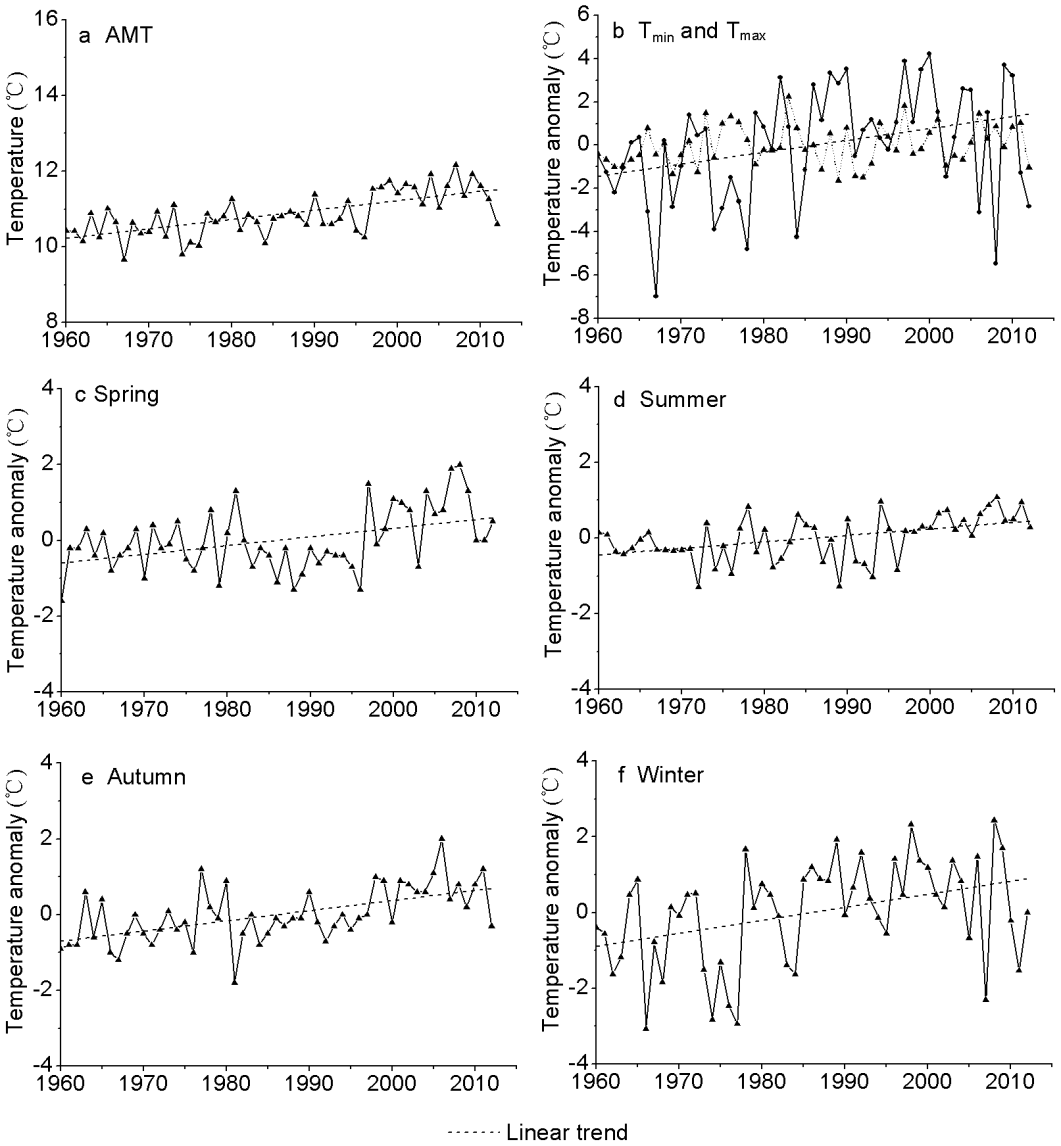
Time series and linear trends of AMT and temperature anomalies in the Tarim Basin. Area averaged (a) AMT, and temperature anomaly for (b) annual minimum (solid line), and maximum (dotted line), (c) spring, (d) summer, (e) autumn, and (f) winter.

We first evaluated the increasing trend of air temperature averaged over the entire basin. There was a significantly linear increase in the AMT (0.25°C/10a, P <0.001) and T_min_ (0.55°C/10a, P = 0.017) (Figure 2, Table 1). While the increasing trend of T_max_ (0.12°C/10a) was not significant, and weaker than that of T_min_, SDDA was smaller for T_max_ (0.9) than for T_min_ (2.43) (Table 1), indicating less fluctuation in T_max_ than in T_min_ relative to their change trends. Trend analyses also showed that there was a significantly (P <0.05) linear increase in the mean temperatures of all seasons, with the greatest in winter (0.36°C/10a), followed by autumn (0.27°C/10a), spring (0.23°C/10a) and summer (0.17°C/10a). However, comparisons of regressions indicated that these trends were not significantly different (P > 0.05, data not shown). SDDA was much smaller in summer than in other seasons, which indicated that temperature fluctuation relative to the trend was smaller in summer than in autumn, winter and spring in the Tarim Basin. Correlation analyses indicated that AMT had a significant relationship (P <0.001) with T_min_ and all seasons' mean temperatures, but no significant relationship with T_max_ (R = 0.26, P = 0.061) (Table 1).

**Table 1:** Trend analyses with regression coefficient (R) and standard deviation of the detrended anomaly (SDDA) of various means of temperature during 1960–2012 and their correlations with AMT.

	AMT	T_max_	T_min_	T_spr_	T_sum_	T_aut_	T_win_
**Trend (°C/10a)**	0.25	0.12	0.55	0.23	0.17	0.27	0.36
**R**	0.668**	0.200	0.328*	0.430**	0.454**	0.565**	0.386**
**SDDA (°C/10a)**	0.43	0.90	2.43	0.74	0.52	0.61	1.26
**R with AMT**		0.259	0.533**	0.564**	0.616**	0.701**	0.335*

Significance of the regression/correlation was marked with one (P <0.05) and two (P <0.01) asterisks.

**Figure 3:**
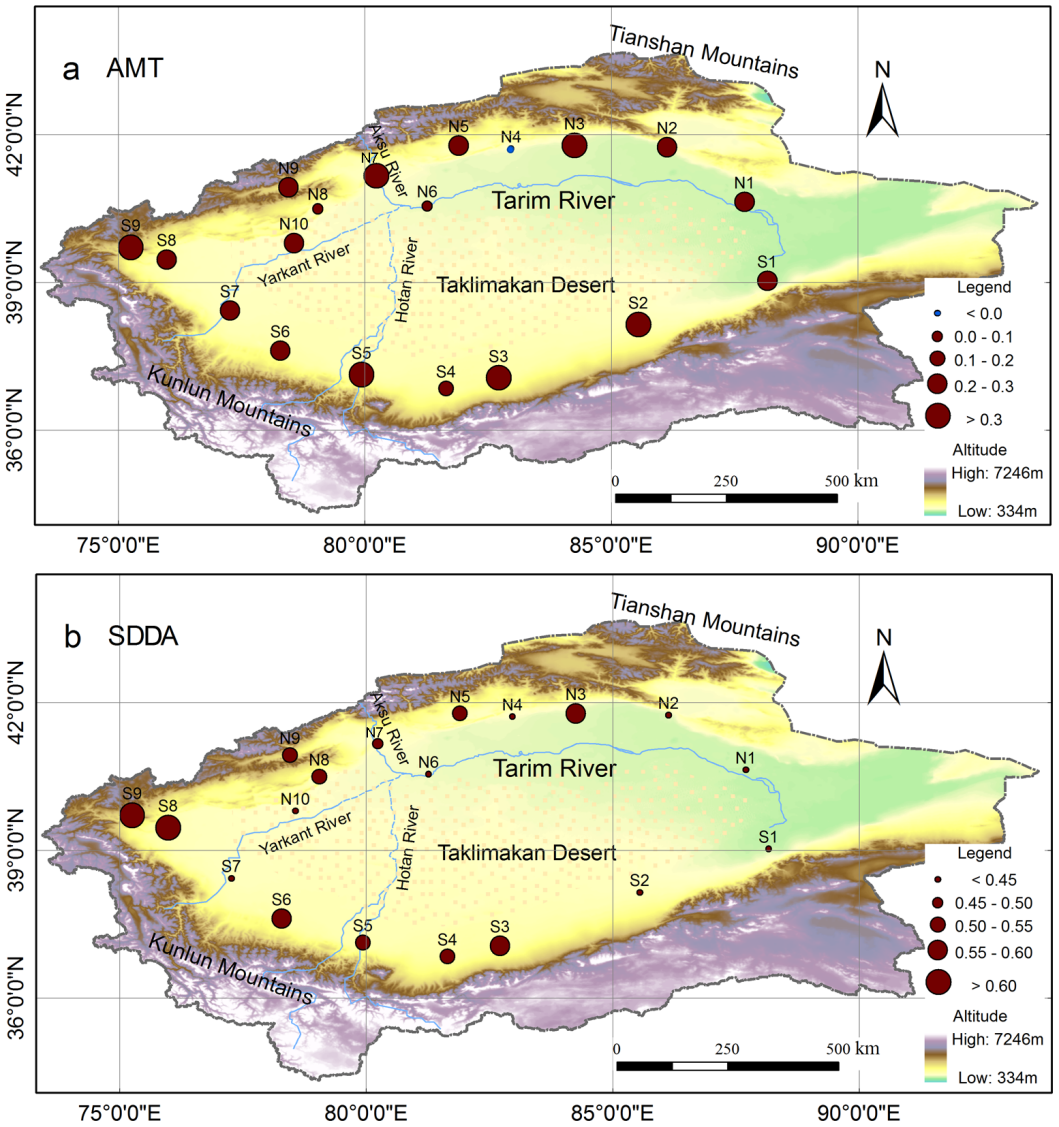
Spatial distributions of linear trend of AMT and its SDDA during 1960–2012 in the Tarim Basin. Spatial distributions of (a) linear trend of AMT (°C/10a) and (b) SDDA (°C/10a) of AMT.

There was a considerably spatial variability in the increasing rate of AMT, ranging from -0.09°C/10a to 0.43°C/10a (Figure 3a), with only one station (i.e., N4) showing a decreasing trend (i.e., −0.09°C/10a). While there seemed no clear spatial pattern, the warming rate of AMT was similar (∼0.26°C/10a) at all the stations located west of 79°E and east of the 86°E. The highest and lowest warming rates were found between 80°E and 86°E. Unlike the warming rate of AMT, the SDDA of AMT revealed a large spatial variation, with much higher values towards the west. In general, SDDA was larger at higher elevation stations than lower elevation stations, indicating that there would be less fluctuation in AMT in the regions with lower elevations.

**Figure 4:**
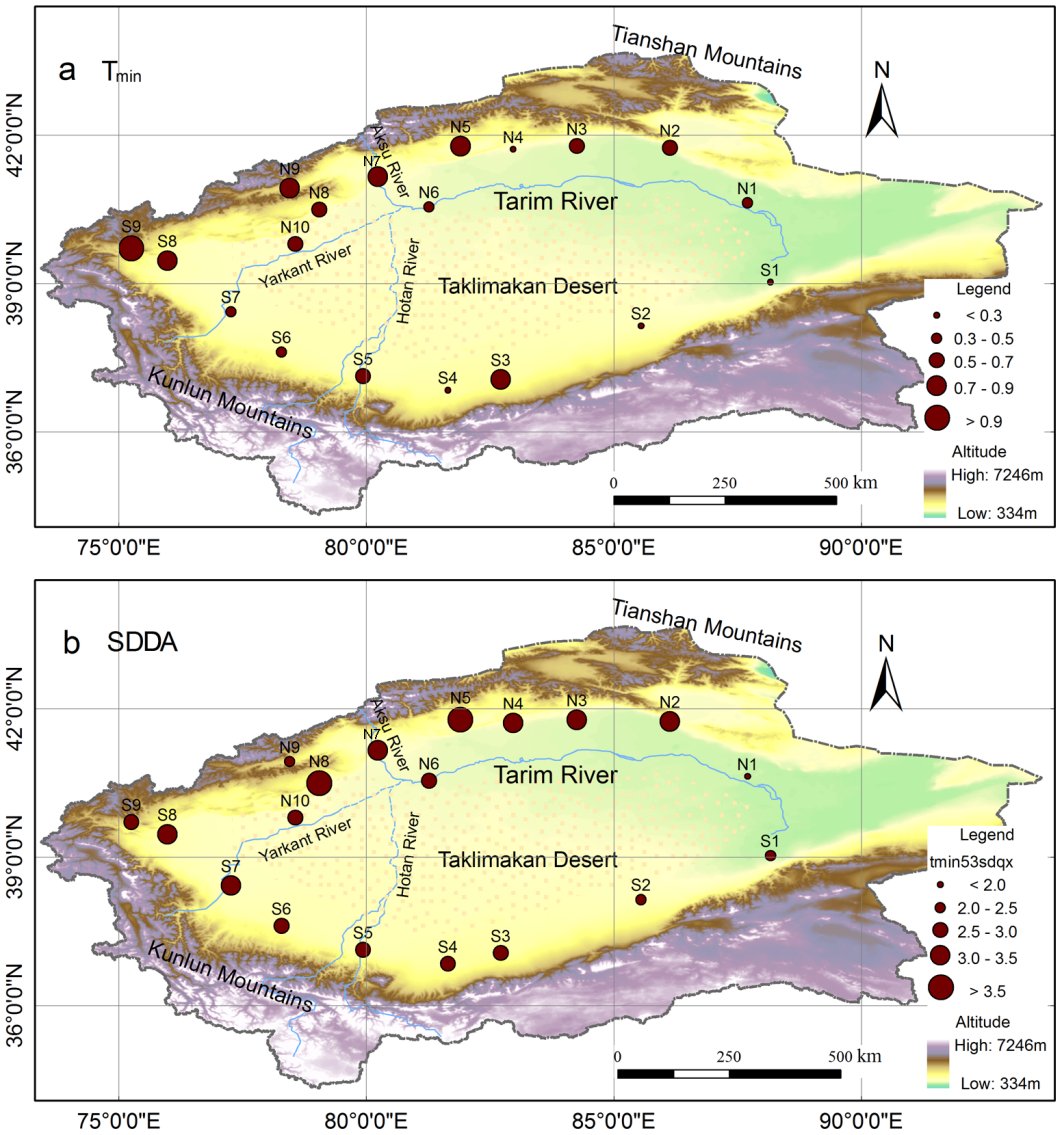
Spatial distributions of linear trend of T_min_ and its SDDA during 1960–2012 in the Tarim Basin. Spatial distributions of (a) linear trend of T_min_ (°C/10a) and (b) SDDA (°C/10a) of T_min_.

Figure 4 shows the spatial pattern of the increasing rate of T_min_ during 1960–2012. There was a big range in the increase of T_min_, i.e., from 0.15 to 1.12°C/10a. In most parts of the Tarim Basin, T_min_ increasing rate reached 0.55°C/10a, which was almost twice as large as the rate of AMT. Clearly, T_min_ increasing rate was significantly higher in the west than in the east. In general, T_min_ revealed a greater increasing rate to the north than to the south, with very high rates (0.80°C/10a) in the most northern edge of the basin. Overall, the SDDA of T_min_ showed a similar spatial pattern to the increasing rate of T_min_, i.e., higher values in the northern parts than in the southern parts, and to the west than to the east of the basin. The exception was at the N4 station that revealed low increasing rate but considerable SDDA for T_min_. These analyses indicated that although the northwest Tarim had greater increasing rate in T_min_, there were larger fluctuations in T_min_ of the northwest stations relative to southeast ones.

### Changes in the seasonal patterns

**Figure 5:**
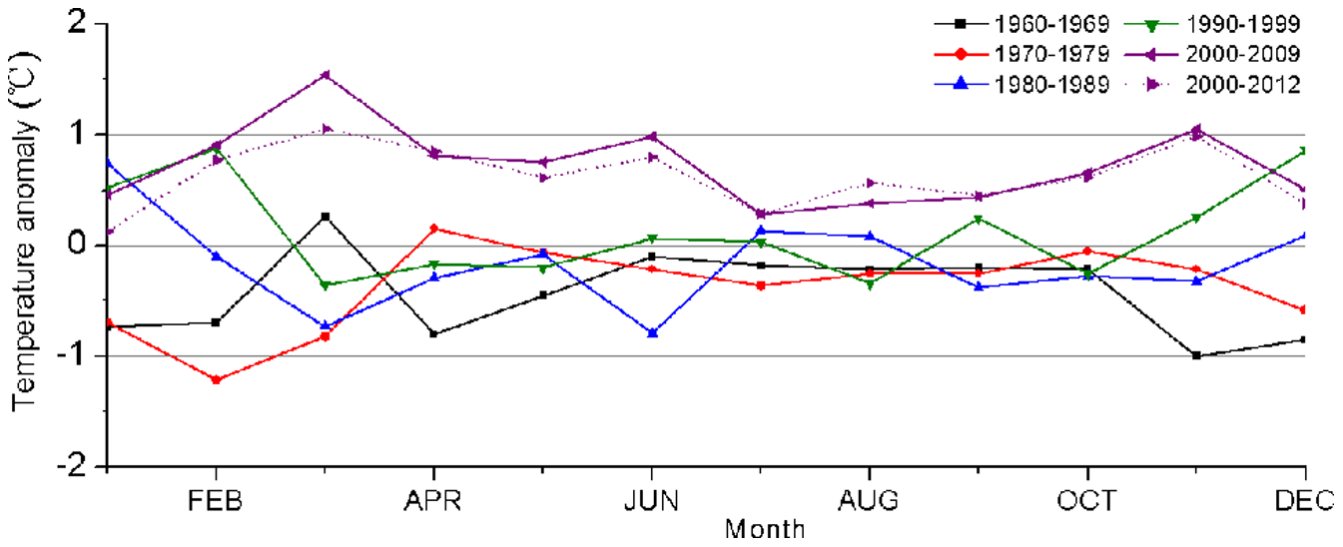
Monthly mean temperature anomalies for different decades in the Tarim Basin.

To understand the seasonal changes, we analyzed the temperature anomalies of individual month for the past five decades (Figure 5). There were large variations between various periods except for the months from July to October. Clearly, air temperature revealed positive anomalies in all the seasons during the most recent decade, with the strongest anomaly found in March (1.54°C) and weakest one in July (0.28°C). On the other hand, there were negative anomalies in most of the months during the period of 1960–1979, with the most negative values found in the winter (−0.6 to −1.21°C). In contrast, the 1980s and 1990s had generally warm winters, with the warmest January in the 1980s and the warmest December in the 1990s. Overall, the largest decadal variability was found in early spring and late autumn.

**Figure 6:**
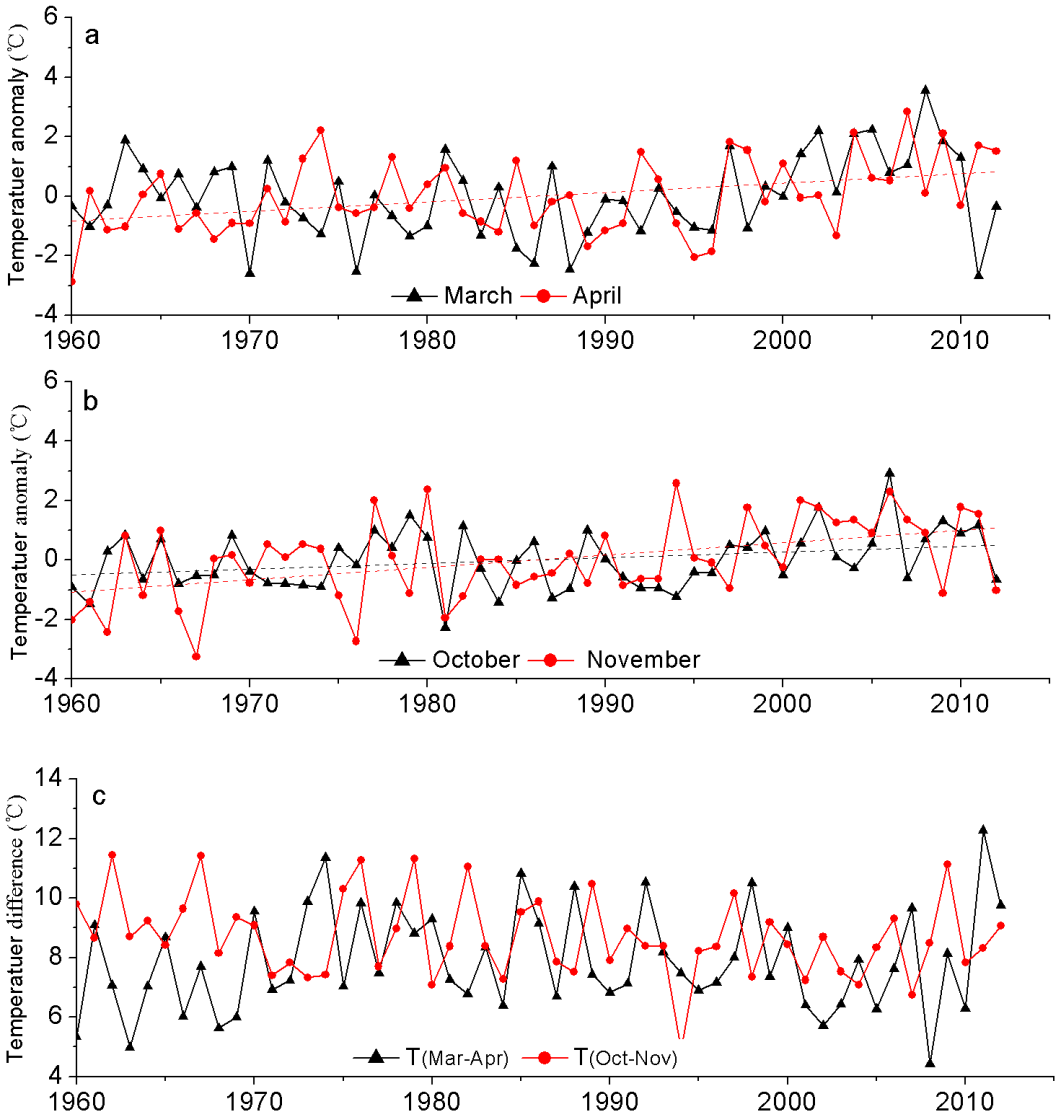
Time series and linear trends of monthly temperature anomalies. Trends for (a) March (black) and April (red), (b) October (black) and November (red), and (c) temperature differences between March and April (black) and between October and November (red).

We further evaluated seasonal change by looking into the temporal variations of monthly temperature anomalies for early spring and late autumn (Figure 6). The temperature anomaly showed an increasing rate of 0.20°C/10a (P = 0.107) in March and 0.32°C/10a (P = 0.004) in April. There was a similarity in temperature anomaly between March and April except during the period of 1960–1976. The temperature anomaly showed a significant increasing trend in October (0.19°C/10a, P = 0.027) and November (0.41°C/10a, P <0.001) from 1960 to 2012. Figure 6c illustrated the temperature increase in spring (i.e., the temperature difference between March and April) and decrease in autumn (i.e., the temperature difference between October and November). Overall, the temperature increase from March to April was much greater post 1970 than prior to 1970 although there was a large year-to-year variability during the most recent five years. Our data showed a general decreasing trend in the temperature change from October to November. For example, average temperature drop was 9.48°C in the 1960s but 8.30°C in the 2000s. These results implied the possibility of shortened spring and autumn with enhanced spring warming and delayed autumn cooling in the Tarim Basin since mid-1970s.

### Relationships with climate indices and local environmental conditions

To explore the possible driving forcing for the temperature change in the Tarim Basin, we carried out correlation analyses, which showed that neither of PDO, SOI and DMI had significantly linear relationship with AMT, T_min_, T_max_ or mean temperature of any season in the Tarim Basin. We also conducted non-linear relationship and multi-regression analyses, but found no significant relationship (data not shown). However, Table 2 showed that the correlation coefficient value was 0.264 (P = 0.059) for the relationship between the spring temperature and the SOI, suggesting that ENSO might have influence on the basin's air temperature in spring. Similarly, there was a weak negative correlation between the PDO index and the AMT in the following year (R = −0.212, P = 0.139), implying that PDO might partly be responsible for the decadal variability of air temperature in the Tarim Basin.

**Table 2:** Correlations between climate indices and various temperature means^a^.

	PDO		SOI^b^		DMI	
	R	*P*	R	*P*	R	*P*
AMT	−0.212	0.139	0.138	0.338	−0.090	0.950
T_max_	0.003	0.984	0.218	0.129	−0.190	0.195
T_min_	−0.066	0.647	0.068	0.640	−0.041	0.784
T_spr_	−0.175	0.214	0.264	0.059	−0.158	0.269
T_sum_	0.110	0.437	0.023	0.874	0.156	0.276
T_aut_	−0.036	0.796	0.036	0.797	0.038	0.791
T_win_	0.166	0.240	−0.144	0.310	−0.176	0.216

a Temperature means for 1961–2012; PDO and DMI were annual means for 1960–2011.

b Mean value over winter (e.g., from December 1960 to February 1961).

Table 3 illustrated that elevation, latitude or longitude had little influence on the warming rate of AMT. However, the warming rate of T_min_ showed significant relationship with elevation (R = 0.535, P = 0.018) and longitude (R = −0.528, P = 0.02), indicating greater warming in T_min_ at higher elevation or west stations. It seems that the significance of combined effects is about the same as that of elevation or longitude.

**Table 3:** Regressions between the warming rate of temperature (AMT and T_min_) and local variables (elevation, latitude and longitude).

	AMT		T_min_	
Variable	R	*P*	R	*P*
Elevation	0.149	0.543	0.535	0.018
Latitude	−0.211	0.385	0.204	0.402
Longitude	−0.010	0.968	−0.528	0.020
Latitude and elevation	0.230	0.648	0.645	0.014
Latitude and longitude	0.210	0.700	0.612	0.023
Longitude and elevation	0.187	0.752	0.588	0.034
Elevation, latitude and longitude	0.255	0.791	0.689	0.019

## Discussion

### Temporal and spatial variations of temperature in the Tarim Basin

In this study, a focus was placed on analyzing spatial and temporal variability of temperature change in the Tarim Basin. Our study showed that there was a large spatial variability (from −0.09 to 0.43°C/10a) in the AMT warming rate during the period 1960–2012 in the Tarim Basin. The spatial variability might be a result of many factors because any single factor such as elevation could not explain the variability. The average rate (0.25°C/10a) in our study was higher than those in previous studies of the Tarim Basin (Table 3), such as 0.20°C/10a during 1960–2001 [21] and 0.22°C/10a during 1960–2007 [11]. The differences in the basin scale mean rate mainly reflected the accelerated warming (0.73°C/10a) in the most recent decade, which was consistent with the analysis by Li, Chen, Shi, Chen and Li [26] who reported a greater temperature increase (0.52°C/10a) for the period of 2000–2010 in northwest China.

An earlier study based on only six stations' data from 1958–2004 in the Tarim Basin indicated that there was a significant temperature increase in autumn and winter but not in spring and summer [15]. However, our analyses using data of 1960–2012 showed a significant warming trend in all four seasons, with the highest increasing rate in winter (0.36°C/10a), and the lowest increasing rate in summer (0.17°C/10a). The difference between these two studies was probably due to the significant warming in spring in the most recent decade (Figure 5), and also reflected the large spatial variability in the Tarim Basin.

Our study also showed that the warming rate was much greater in T_min_ (0.55°C/10a) than in T_max_ (0.12°C/10a) during the period 1960–2012, and there was a significant correlation between AMT and T_min_. These analyses indicated that air temperature increase in winter was largely responsible for the AMT increase. Overall, the seasonality had become weaker, particularly during the period of 1975–2009 in the Tarim Basin. Further analyses seemed to show an enhancement of the early spring warming and a reduction of the late autumn cooling. In addition, the increasing rate in winter (0.36°C/10a) was greater than in summer (0.17°C/10a). All these might be attributable to the weakening of the seasonality in the Tarim Basin.

### Comparison between the Tarim Basin and other regions

The global mean surface air temperature had risen by 0.74±0.18°C/100y during the twentieth century and was projected to rise by 1.8–4.0°C in the twenty-first century [4], [27]. For the period of 1880–2003, the linear increase of AMT over China was 0.58°C/100y, which was slightly weaker than that of the global mean [28]. However, the warming rate (0.30°C/10a) during the past two decades in China was much stronger than that of the global mean (0.19°C/10a) [29], [30]. Table 4 illustrated that warming rate of the AMT was greater than 0.34°C/10a in the northwest China, but less than 0.3°C/10a in Xinjiang. Limited studies, including our study, indicated that on average, warming trend in the Tarim Basin was not as strong as that of China, which might be attributable to the smaller increase of temperature in summer.

**Table 4:** Increasing trends in mean air temperatures at various spatial and temporal scales.

Region	Stations	Period	Trend	References
**Global**		1901–2000	0.6°C/100a	[46]
**Global**		1906–2005	0.74°C/100a	[47]
**Global**		1956–2005	0.13°C/10a	[47]
**China**	726	1951–2004	0.25°C/10a	[8]
**China**	726	1905–2001	0.5–0.8°C/100a	[47]
**Northern China**	486	1960–2000	0.2–0.3°C/10a	[48]
**Southern China**	486	1960–2000	<0.1°C/10a	[49]
**Northwest China**	135	1960–2005	0.37°C/10a	[13]
**Northwest China**	138	1961–2006	0.35°C/10a	[9]
**Northwest China**	74	1960–2010	0.343°C/10a	[49]
**Xinjiang**	77	1955–2000	1°C/50a	[50]
**Xinjiang**	65	1961–2005	0.28°C/10a	[10]
**Xinjiang**	50	1961–2008	0.30°C/10a	[51]
**Tarim**	26	1960–2001	0.20°C/10a	[52]
**Tarim**	23	1959–2006	0.1–0.4°C/10a	[53]
**Tarim**	25	1960–2007	0.22°C/10a	[11]
**Tarim**	13	1957–2005	0.19°C/10a	[12]
**Tarim**	19	1960–2012	0.25°C/10a	This study

Some studies showed an important feature associated with climate warming, i.e., the asymmetric nature over the daily cycle, with less warming observed in daily maximum temperature than in daily minimum temperature [31], [32], especially in winter. A similar feature was also found over the seasonal cycle in many regions. For example, Turkes and Sumer [33] reported a significant warming of T_min_ but a weak warming and/or cooling in T_max_ in many regions of Turkey. Studies in China revealed stronger warming in T_min_ (0.32°C/10a) than in T_max_ (0.13°C/10a) [34], [35]. While the increasing rate of T_max_ was comparative, the increasing rate of T_min_ was much greater in the Tarim Basin (0.55°C/10a). Similarly, based on data from 19 synoptic stations in the arid and semi-arid regions of Iran for the period of 1966–2005, Tabari [36] found that the increasing trends in the T_min_ series (0.44°C/10a) were much stronger than those in the T_max_ series (0.09°C/10a). These results imply that under the global warming, arid and semi-arid regions have less extremely cold days in winter.

### Impacts of local environments and large scale climate phenomena

There was evidence of warming rate increasing with the increase of latitude and/or elevation [10], [37], [38]. However, some studies showed a reduction in the warming rate at high elevations [39], [40] or lack of clear relationship between the warming rate and elevation [41]. At the global scale, there was no simple relationship between elevation and warming rate [42]. Our analyses showed that the warming rate of AMT had little relationship with elevation (R = 0.149, P = 0.543) and latitude (R = −0.211, P = 0.385) in the Tarim Basin. However, there was a significant correlation between the warming rate of T_min_ and elevation (R = 0.535, P = 0.018), indicating greater increase of T_min_ at higher elevation. The facts of significant relationship between AMT and T_min_ and great warming rate of T_min_ over 1960–2012 suggest that elevation may have an influence on the basin scale warming trend.

There have been limited studies that addressed the impacts of the ENSO phenomenon on the temperature in the Tarim Basin, showing inconsistent conclusions [15], [17]. Using the latest dataset, our analyses indicated that ENSO events (mainly during December-February) would affect the spring temperature. Further analyses showed that there were lagged effects of both ENSO and PDO on air temperature of the Tarim Basin, i.e., ENSO on the temperature in May (R = 0.392, P = 0.004) and PDO on the temperature in July (R = 0.308, P = 0.026) of the following year.

**Figure 7:**
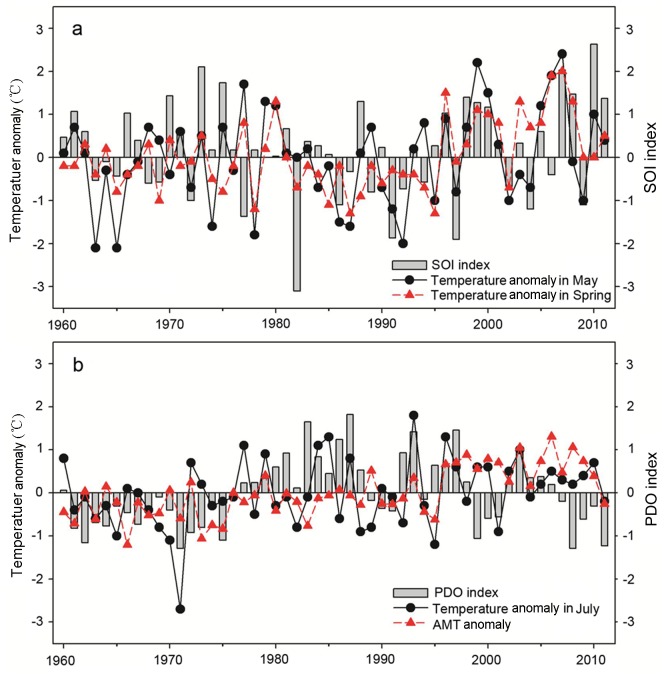
Time series of temperature anomalies and SOI and PDO indices in the Tarim Basin. Time series of (a) SOI and temperature anomalies in spring and May, and (b) AMT anomaly, temperature anomaly in July and PDO index.

Figure 7 illustrated that there was a large similarity in the temporal variability for the SOI, mean temperature in May and in spring, particularly post the early 1980s. In addition, the cold ENSO phases (with positive SOI, e.g., 1998–2000, and 2007) during the most recent two decades corresponded with extremely warm temperature in May in the Tarim Basin. It seemed that there was a positive correlation between the PDO index and mean air temperature in July prior to the mid-2000s. For example, both showed an increasing trend from the early 1970s to mid-1980s, indicating a warming trend. However, for the period post late 1990s, PDO revealed mainly cold phases whereas positive anomalies (i.e., strong warming) were seen for both AMT and temperature in July.

There was evidence that the long-term climate variations in winter and summer in China might be connected to the warming trend in the sea surface temperature of the Indian Ocean [43]. Recent studies also indicated that decadal to interdecadal variability in the climate changes in Asia might be related to other climatic phenomena, such as the Arctic Oscillation [44], and Asia-Pacific Oscillation [45]. All these analyses indicated that the relationship between any of the climate indices and air temperature in the Tarim Basin might be non-linear, implying complex impacts of multi factors associated with local environmental driving and remote forcing.

### Conclusions and implications

This study demonstrated a significantly increasing trend in air temperature in all four seasons during 1960–2012 in the Tarim Basin. However, there was large spatial and temporal variability in the warming rate. Temperature increase was much greater in the T_min_ (0.55°C/10a) than in AMT (0.25°C/10a) and T_max_ (0.12°C/10a), and warming rate was 0.36, 0.27, 0.23 and 0.17°C/10a in winter, autumn, spring and summer, respectively. The warming was most pronounced in the most recent decade, which might be associated with both PDO and ENSO phenomena that were in cold phases.

There was an overall weakening in the seasonality of air temperature since mid-1970s in the Tarim Basin. Apart from being less cold in winter, spring warming was another feature, which would have impacts on the hydrological cycle in the basin. Particularly, increasing temperature would lead to enhanced melting of snow and glacier in the surrounding mountains, causing extreme runoff events such as floods with a wide range of implications. Future studies are in need to better understand the climate change at various spatial scales and underlying mechanisms, and also to assess the impacts of climate change on environmental and economic aspects.
